# Complete Genome Sequence of Nonhemolytic *Streptococcus agalactiae* Serotype V Strain 1, Isolated from the Buccal Cavity of a Canine

**DOI:** 10.1128/genomeA.01612-15

**Published:** 2016-01-28

**Authors:** Leeanne K. Harden, Karina M. Morales, Jeffery R. Hughey

**Affiliations:** Division of Mathematics, Science and Engineering, Hartnell College, Salinas, California, USA

## Abstract

The complete genome sequence from a nonhemolytic strain of *Streptococcus agalactiae* from the oral cavity of a canine was assembled. The genome is 2,165,968 bp, contains 2,055 genes, and is classified as group B streptococcus (GBS) serotype V, strain 1. A comparison to other *S. agalactiae* sequences shows high gene synteny with human and bovine strains.

## GENOME ANNOUNCEMENT

*Streptococcus agalactiae* (group B streptococcus [GBS]) is a common Gram-positive colonizer of terrestrial and aquatic vertebrates, including humans, cows, rabbits, cats, dogs, horses, frogs, trout, dolphins, and seals (1, 2). GBS is a leading cause of invasive infections in neonates (1) and is implicated in a number of animal diseases, including bovine mastitis (3). Characterization of the genetic diversity of GBS within the oral microbiota of animals, especially pets that interact closely with humans, is critical to understanding better the source of opportunistic pathogens and to documenting bacterial communities. Ten serotypes are currently described for *S. agalactiae* (4); here, we report the complete genome sequence of *S. agalactiae*, serotype V, strain 1 (ST-1), isolated from the buccal cavity of *Canis lupus familiaris*.

Genomic analysis of *S. agalactiae* ST-1 was performed using Illumina 36-bp paired-end sequencing methods and resulted in 23,645,928 reads. The data were assembled with default *de novo* settings in CLC Genomics Workbench (Qiagen), with an average coverage of 345×. Gaps were closed by mapping in Geneious version 8.1.5 (Biomatters Ltd., San Francisco, CA) against GenBank accession numbers CP010867 and HF952104, and by using standard PCR and Sanger sequencing. The genome was annotated using the NCBI Prokaryotic Genome Annotation Pipeline 3.0 with GeneMarkS+ (http://www.ncbi.nlm.nih.gov/genome/annotation_prok/).

*S. agalactiae* ST-1 has a chromosome length of 2,165,968 bp. The genome contains 2,055 protein-coding genes (CDSs), 80 tRNAs, and 21 rRNAs, with 7 copies each of 16S, 23S, and 5S rRNA genes. It also includes 3 prophage-like elements and 1 clustered regularly interspaced short palindromic repeat. The genome is most closely related to *S. agalactiae* strains isolated from the blood of a human, *S. agalactiae* SS1 (1), and from bovine milk from a cow with mastitis, *S. agalactiae* 09mas018883 (3). These two strains differ in genetic distance from the canine *S. agalactiae* ST-1 by 0.016 and 0.027, respectively. Alignment of the sequences against *Streptococcus dysgalactiae* (accession no. AP011114) using MAUVE version 2.3.1 (5) showed high gene synteny for the three genomes of *S. agalactiae*, supporting the pangenome proposal of Tettelin et al. (6). The alignment identified 129 locally collinear blocks (LCBs), of which three LCBs were unique to *S. agalactiae* ST-1. The LCBs were 9,662 bp (containing 9 genes), 12,794 bp (17 genes), and 50,260 bp (58 genes) in length. The LCB genes were predominantly hypothetical proteins. A single large LCB of 46,595 bp in length (47 genes) is present in *S. agalactiae* 09mas018883 but was not found in *S. agalactiae* ST-1 or *S. agalactiae* SS1. Analysis of the *S. agalactiae* ST-1 genome using IslandViewer 3 (7) found 14 genomic islands; however, no virulence, resistance, or pathogen-associated genes were predicted.

Although *S. agalactiae* is reported in canines (8), several extensive genetic analyses of the canine oral microbiome identifying >350 taxa failed to identify *S. agalactiae* (9–11). These data represent the first record of *S. agalactiae* isolated from the oral cavity of a dog and suggest that further work is needed to fully characterize the canine oral microbiome.

### Nucleotide sequence accession number.

This whole-genome shotgun project has been deposited in GenBank under the accession number CP013202. The version described in this paper is the first version.
